# Analysis of independent risk factors for high hidden blood loss following total knee arthroplasty

**DOI:** 10.1371/journal.pone.0356264

**Published:** 2026-09-03

**Authors:** Kaige Xu, Weipeng Shi, Tianyu Guo, Jiaqi Dong, Tianlong Gao, Yaping Jiang, Tao Li

**Affiliations:** 1 Department of Joint Surgery, The Affiliated Hospital of Qingdao University, Qingdao, China; 2 Department of Oral Implantology, The Affiliated Hospital of Qingdao University, Qingdao, China; 3 School of Stomatology, Qingdao University, Qingdao, China; Stanford University School of Medicine, UNITED STATES OF AMERICA

## Abstract

**Background:**

Hidden blood loss (HBL) constitutes a substantial proportion of total perioperative blood loss after total knee arthroplasty (TKA) and may adversely affect postoperative recovery, yet factors associated with high-level HBL remain insufficiently defined. This study aimed to investigate potential risk factors and the predictive value of high HBL following TKA.

**Methods:**

A retrospective cohort study was conducted at The Affiliated Hospital of Qingdao University. We included 154 patients undergoing primary unilateral TKA for knee osteoarthritis in 2024. Participants were categorized into low HBL (<654 mL) and high HBL (≥654 mL) groups. Data were analyzed using Student’s t-test, Mann-Whitney U test, and Chi-square test as appropriate. Multivariate binary logistic regression was performed to identify independent risk factors, with multicollinearity assessed via VIF. Predictive performance was evaluated using ROC curve analysis and further validated through 10-fold cross-validation in R. A two-sided P < 0.05 was considered statistically significant.

**Results:**

Body mass index (BMI) emerged as an independent risk factor for high HBL (OR = 1.306; 95% CI, 1.147–1.487; P < 0.001). Preexisting hypertension was also associated with increased risk (OR = 2.986; 95% CI, 1.278–6.974; P = 0.011). In predictive analyses, BMI demonstrated superior discriminative ability (AUC = 0.778) compared with hypertension (AUC = 0.622), with an optimal cut-off value of 26.34 kg/m².

**Conclusion:**

Elevated BMI (>26.34 kg/m²) was an independent predictor of high HBL following TKA, whereas preexisting hypertension represented an associated risk factor. Routine preoperative assessment of BMI could facilitate early identification of patients at high risk, enabling individualized perioperative blood management. Integrating BMI evaluation into surgical planning allowed clinicians to offer targeted weight optimization counseling and implement tailored blood-conservation strategies, ultimately reducing HBL and enhancing postoperative recovery and patient safety.

## Introduction

Knee osteoarthritis (KOA) is the most prevalent musculoskeletal disorder among individuals over 60 years old and represents a leading cause of chronic pain and functional limitation [1–3]. Total knee arthroplasty (TKA) remains the definitive treatment for end-stage KOA, offering substantial pain relief and marked improvement in joint function [4–6]. Nevertheless, the procedure involves extensive soft tissue release and bone osteotomy, which can result in considerable intraoperative and postoperative blood loss [7]. Excessive bleeding may prolong hospitalization, delay functional recovery, and increase transfusion requirements, each of which carries additional risks, including infection, thromboembolic events, and higher healthcare costs [8–12].

In addition to visible intraoperative and postoperative drainage loss, a substantial but often overlooked component of total blood loss after TKA is hidden blood loss (HBL), defined as blood sequestration within tissues and joint cavities that is not directly measurable during surgery. Since being first reported by Sehat et.al, HBL has been increasingly recognized as accounting for a significant proportion of perioperative blood loss in arthroplasty patients and has been associated with postoperative anemia and delayed recovery [13–15]. Although the routine use of tranexamic acid (TXA) has significantly reduced visible blood loss and transfusion rates, HBL remains the predominant component of residual perioperative blood loss. Recent studies have shown that HBL still accounts for more than half of total blood loss in TXA-treated patients, underscoring its ongoing clinical importance [16,17].

With the growing demand for TKA, identifying factors that predispose patients to elevated HBL has become a critical clinical priority. Previous investigations have implicated factors such as gender, age, and hypertension as potential contributors to increased HBL [18,19]. Notably, a retrospective study of 1,444 primary TKA cases specifically targeted the “high-level” HBL subgroup, identifying coronary artery disease, lower preoperative platelet counts, conventional mechanical alignment, and longer operation times as significant risk factors [20]. However, despite this valuable contribution, comprehensive literature specifically targeting “high-level” HBL remains scarce, as the vast majority of existing studies continue to focus primarily on overall or mean blood loss.

Although HBL has received increasing attention after total knee arthroplasty, no universally accepted clinical threshold for high-level HBL has been established. Many previous studies have focused primarily on mean HBL values or factors associated with overall blood loss, rather than applying a standardized criterion to identify patients with relatively high HBL [21–23]. Mean-based cutoff values may be influenced by cohort characteristics and may lack reproducibility. Therefore, the upper quartile of the HBL distribution was used in the present study as an objective distribution-based criterion for defining high HBL. We retrospectively analyzed 154 patients who underwent TKA at our institution between January and December 2024 to identify factors independently associated with high HBL and to evaluate the discriminative performance of significant factors. We hypothesized that selected patient and perioperative characteristics would be associated with membership in the upper-quartile HBL subgroup.

## Materials and Methods

### Study setting and design

This study was conducted at the Affiliated Hospital of Qingdao University, a tertiary referral center for joint arthroplasty. We performed a retrospective observational cohort study to investigate factors associated with HBL following primary unilateral TKA. Clinical and laboratory data were extracted from the hospital’s electronic medical record system for eligible patients treated between January 10 and December 31, 2024.

### Participants and sample size

A total of 154 patients who underwent primary unilateral TKA for knee osteoarthritis (KOA) were included in the final analysis，comprising 42 male and 112 female patients, with a mean age of 66.12 ± 6.07 years (range, 50–85 years). Inclusion criteria were as follows: (1) patients diagnosed with KOA who had failed to respond to conservative treatment; (2) patients undergoing unilateral primary TKA with no prior surgical intervention on the affected knee; (3) absence of recent surgical or invasive procedures; and (4) availability of complete preoperative laboratory and diagnostic data. Exclusion criteria included: (1) hematologic disorders affecting coagulation, such as thrombocytopenia, hemophilia, or thrombosis; (2) recent use of anticoagulant medications or blood transfusion within the preceding 3 months; (3) coexisting malignancies or systemic conditions impacting coagulation function, including liver or renal disease; (4) psychiatric or neurological comorbidities that could interfere with postoperative management; and (5) incomplete clinical records.

### Study instruments, tools, and procedures

#### Surgical technique and perioperative management.

All surgical procedures were performed by the same senior surgeon following a standardized operative protocol to ensure procedural consistency and reproducibility. All patients received posterior-stabilized (PS) prostheses with the identical bone cement fixation techniques. General anesthesia was administered, and patients were positioned supine. A pneumatic tourniquet was applied at a pressure of 280–300 mmHg. In the full-tourniquet group, inflation was maintained from skin incision until complete wound closure. In the partial-tourniquet group, the tourniquet was inflated only from the time of bone cement implantation until wound closure. A midline anterior approach to the knee, combined with a medial parapatellar incision, was utilized to access the joint capsule. Soft tissues, including the infrapatellar fat pad and hypertrophic synovium, were excised, and the medial collateral ligament insertion was released as necessary. The distal femur was prepared using intramedullary alignment with 3° external rotation osteotomy, while the tibia was prepared with extramedullary alignment at an appropriate posterior slope. After balancing the joint gap, trial components were inserted, the patella was reshaped, and selective nerve ablation was performed using electrocautery. The joint was thoroughly irrigated, followed by fixation of the components with bone cement. Once the cement had set, the joint was irrigated again, and the capsule was closed. Local anesthetic was infiltrated into the surrounding soft tissues. The decision to place a drainage tube was left to the operating surgeon’s discretion based on intraoperative assessment.

**Tranexamic acid(TXA) protocol:** All patients received a standardized TXA regimen. Intravenous TXA (1 g) was administered at the induction of anesthesia, followed by intra-articular TXA (1.5 g) prior to wound closure. No additional antifibrinolytic agents were employed.

**Antiplatelet and anticoagulant management:** Patients on aspirin, clopidogrel, or other antiplatelet agents discontinued these medications 7 days prior to surgery. Postoperatively, rivaroxaban (10 mg once daily) was prescribed starting on postoperative day 1 for venous thromboembolism prophylaxis.

**Management of comorbidities:** Common comorbidities, including hypertension, diabetes mellitus, and coronary artery disease, were routinely evaluated and managed according to institutional perioperative protocols and current clinical guidelines. For patients with a history of hypertension, antihypertensive medications were continued throughout the perioperative period unless contraindicated. Blood pressure was monitored regularly, and surgery was performed only when preoperative blood pressure was adequately controlled. Patients with uncontrolled hypertension or acute cardiovascular instability were deferred until stabilization was achieved. No patient underwent surgery under hypertensive crisis conditions. During hospitalization, blood pressure fluctuations were managed by the anesthesiology and medical teams using standardized protocols, aiming to minimize hemodynamic instability that could potentially influence perioperative blood loss.

**Baseline anemia definition:** Preoperative anemia was defined according to WHO criteria as hemoglobin (Hb)<130 g/L in men or <120 g/L in women, or hematocrit (Hct)<39% in men and <36% in women.

**Postoperative care:** Postoperative management included bandage compression, limb elevation, and the application of cold compresses. Drains were initially clamped for 2 hours following surgery and then opened; removal was performed when the daily drainage volume fell below 50 mL. Analgesia was provided through a combination of intravenous and oral opioids. Prophylactic antibiotics and anticoagulation therapy were initiated on the first postoperative day. Patients were instructed to perform daily ankle pump exercises, and assisted ambulation was encouraged starting on postoperative day 2.

### Observational indicators and measurement tools

Patient demographics and perioperative data were extracted from the institution’s electronic medical record system, including age, sex, body mass index (BMI), preoperative knee range of motion (ROM), and comorbidities such as hypertension. ROM was measured with patients in the supine position as the angle formed between the longitudinal axes of the femur and tibia at the extremes of active flexion and extension. Hypertension was defined as a documented prior diagnosis, current use of antihypertensive medications, or a measured systolic blood pressure ≥140 mmHg or diastolic blood pressure ≥90 mmHg on at least two separate occasions prior to surgery. Fasting venous blood samples were collected on the morning of the 2nd day after admission, as well as on the second and fourth postoperative mornings. Haemoglobin (Hb) concentration and haematocrit (Hct) were analyzed in the hospital central laboratory using standard automated methods.

Calculation of HBL: (1)according to the formula of Nadler [24]: Predicted Blood Volume (PBV)  =  k1  ×  height(m)3  +  k2 × weight (kg)  +  k3 (for male: k1  =  0.3669, k2  =  0.03219, and k3  =  0.6041; for female: k1  =  0.3561, k2  =  0.03308, and k3  =  0.1833).(2)According to the Gross formula [25]: total blood loss (TBL)  =  PBV (Hct_pre_  −  Hct_post_)/Hct_ave_, where Hct_pre_ is the preoperative Hct, Hct_post_ is the Hct measured on the morning of postoperative day 2 or day 4, and Hct_ave_ is the average of Hct_pre_ and the Hct_post_.(3)Visible blood loss(VBL)= Intraoperative suction canister volume- Subtract irrigation fluid volume+ Net increase in gauze weight+Cumulative drainage volume  .(4)HBL  =  TBL – VBL. All patients in this study did not receive perioperative blood transfusions; therefore, transfusions were not included in the HBL calculation.

**Definition of high HBL:** Because no universally accepted clinical cutoff for high HBL following TKA has been established, a quartile-based classification approach was adopted to provide an objective and reproducible grouping criterion. The 75th percentile (Q3) of the HBL distribution was 654 mL. Patients with HBL ≥ 654 mL were classified into the highest quartile group (Q4, high-HBL group), whereas those with HBL < 654 mL were classified into the low-HBL group.

### Ethical approval

All procedures performed in studies involving human participants were in accordance with the ethical standards of World Medical Association Declaration of Helsinki Ethical Principles for Medical Research Involving Human Subjects. All methods were approved by the Ethics Committee of the Affiliated Hospital of Qingdao University(Ethical Approval Number: QYFY WZLL30453). Written informed consent was obtained from all subjects and/or their legal guardian(s).

### Statistical analysis

All statistical analyses were conducted using SPSS version 26.0 (IBM Corp., Armonk, NY, USA). Continuous variables were evaluated for normality using the Shapiro–Wilk test and visual inspection of Q-Q plots. For clinical intuitiveness, all continuous variables are presented as mean ± standard deviation (SD). Group comparisons for normally distributed variables were performed using Student’s t-test. For variables that exhibited a skewed distribution, the Mann-Whitney U test was employed to ensure the robustness of the results, as it does not require the assumption of normality. Categorical variables are expressed as frequencies and percentages, and comparisons between groups were conducted using the chi-square test or Fisher’s exact test, as appropriate.

Independent predictors of high HBL were identified using binary logistic regression, with multicollinearity among variables assessed via variance inflation factor (VIF). The diagnostic performance of significant factors was evaluated using receiver operating characteristic (ROC) curve analysis, and optimal cutoff points were determined using the Youden index. Missing data were handled via complete-case analysis. In addition, internal validation of the ROC analysis for BMI was performed in R (version 4.3.0; R Foundation for Statistical Computing, Vienna, Austria) using 10-fold cross-validation to estimate the area under the curve (AUC) and optimal cutoff value. A two-sided p-value <0.05 was considered statistically significant.

## Results

### Clinical outcomes

All patients successfully underwent surgery without intraoperative complications or the need for postoperative blood transfusion. In the low-HBL group (n = 116), the mean HBL was 330.00 ± 171.67 mL. In the high-HBL group (n = 38), the mean HBL was 972.95 ± 243.29 mL. To validate the clinical relevance of the 654 mL threshold, a post-hoc analysis was performed, which revealed that the high-HBL group experienced a significantly greater reduction in hemoglobin levels compared to the low-HBL group (P < 0.001; Table 1).Despite the markedly greater blood loss observed in the high-HBL group, no patient required transfusion, as postoperative Hb levels remained above the institutional threshold of 70 g/L or did not meet criteria for symptomatic anemia.

**Table 1:** Univariate Analysis of Factors Associated with HBL.

Variables		Low HBL	High HBL	*t/*χ*²*	P
Age(year)		66.37 ± 6.07 (65.25,67.49)^1^	65.34 ± 6.095 (63.34,67.35)	0.906	0.367^4^
Gender(%)				2.329	0.144
	Female	88(75.9)^2^	24(63.2)		
	Male	28(24.1)	14(36.8)		
BMI(kg/m²)		25.57 ± 3.51 (24.93,26.22)	28.67 ± 2.76 (27.77,29.58)	–	0.000*
Surgical side(%)				−0.424	0.577
	Left	54(46.6)	20(52.6)		
	Right	62(53.4)	18(47.4)		
Operative time (min)		73.32 ± 9.77 (71.52,75.11)	77.76 ± 9.12 (74.77,80.76)	–	0.013*
Tourniquet(%)				1.452	0.310
	Partial-course	8(6.9)	5(13.2)		
	Full-course	108(93.1)	33(86.8)		
Drainage tube(%)				0.073	0.851
	no	67(57.8)	21(55.3)		
	yes	49(42.2)	17(44.7)		
Preoperative ROM^3^(°)		105.82 ± 16.47 (102.79,108.85)	104.87 ± 12.55 (100.74,108.99)		0.394^4^
Preoperative hemoglobin(g/L)		132.41 ± 13.75 (129.88,134.94)	135.55 ± 12.53 (131.43,139.67)		0.132^4^
Hypertension(%)				6.841	0.014*
	no	68(58.6)	13(34.2)		
	yes	48(41.4)	25(65.8)		
Diabetes(%)				0.001	1.000
	no	104(89.7)	34(89.5)		
	yes	12(10.3)	4(10.5)		
Hemoglobin Reduction (g/L)		22.72 ± 7.85	40.86 ± 8.78		0.000*^4^

*,The intergroup differences were statistically significant (P < 0.05); ^1^, 95% confidence interval(CI) for this continuous variable;^2^,Percentage of the reorganized total accounted for by this count data;^3^,ROM, knee joint range of motion;^4^, Analyzed using the Mann-Whitney U test due to non-normal distribution (includes BMI, Operative time, Preoperative ROM, and Hemoglobin Reduction); all other continuous variables were compared using Student’s t-test.

### Univariate analysis

Univariate analyses were conducted on 11 variables, including age, sex, BMI, operative time, and other perioperative factors. Three factors were found to be significantly associated with an increased risk of high postoperative HBL (p < 0.05): BMI (P = 0.000), operative time (P = 0.013), and the presence of hypertension (χ² = 6.841, P = 0.014). The remaining eight variables did not show statistically significant differences between groups (all P > 0.05). Detailed results are presented in Table 1.

### Binary logistic regression analysis

The three factors identified as significant in univariate analysis, BMI, hypertension and operative time, were included in a binary logistic regression model to determine independent predictors of high postoperative HBL. An interaction term between BMI and hypertension (BMI × hypertension) was also incorporated into the model. The interaction term was not statistically significant (β = 0.096, P = 0.475), indicating no detectable interaction effect between BMI and hypertension.

Subsequently, BMI (β = 0.267; OR = 1.306; 95% CI: 1.147–1.487; P < 0.001) and hypertension (β = 1.094; OR = 2.986; 95% CI: 1.278–6.974; P = 0.011) were confirmed as independent risk factors for high postoperative HBL (Table 2). The Hosmer–Lemeshow goodness-of-fit test showed a statistically significant discrepancy between observed and predicted probabilities (χ² = 17.707, df = 8, P = 0.024), indicating limited calibration of the logistic regression model (Table 2).

**Table 2:** Logistic Regression Analysis of Factors Associated with HBL.

Variables	β	Standard Error	Wald	OR^3^	95%CI^3^	P
BMI	0.267	0.066	16.301	1.306	(1.147,1.487)	0.000^1^
Operative time	0.024	0.022	1.163	1.024	(0.981,1.069)	0.281
Hypertension	1.094	0.433	6.388	2.986	(1.278,6.974)	0.011^1^
BMI*Hypertension	0.096	0.135	0.510	1.101	(0.845,1.434)	0.475^2^

^1^, P < 0.05 was considered statistically significant;^2^ The interaction term (BMI × Hypertension) was included in the model but was not statistically significant (P = 0.475); ^3^, OR odds ratio; CI confidence interval. The Hosmer–Lemeshow test suggested limited calibration of the logistic regression model (χ² = 17.707, df = 8, P = 0.024).

### Multicollinearity assessment

Potential multicollinearity among BMI, hypertension, and operative time was evaluated using VIF. All VIF values were approximately to 1, indicating the absence of multicollinearity and confirming that the independent variables were suitable for inclusion in the regression model (Table 3).

**Table 3:** VIF For Variables in Logistic Regression.

Variables	Tolerance	VIF*
BMI	0.912	1.097
Hypertension	0.982	1.019
Operative time	0.896	1.116

*,VIF values <5 were considered to indicate no substantial multicollinearity

### Predictive value of BMI and hypertension for high postoperative HBL

**Fig 1:**
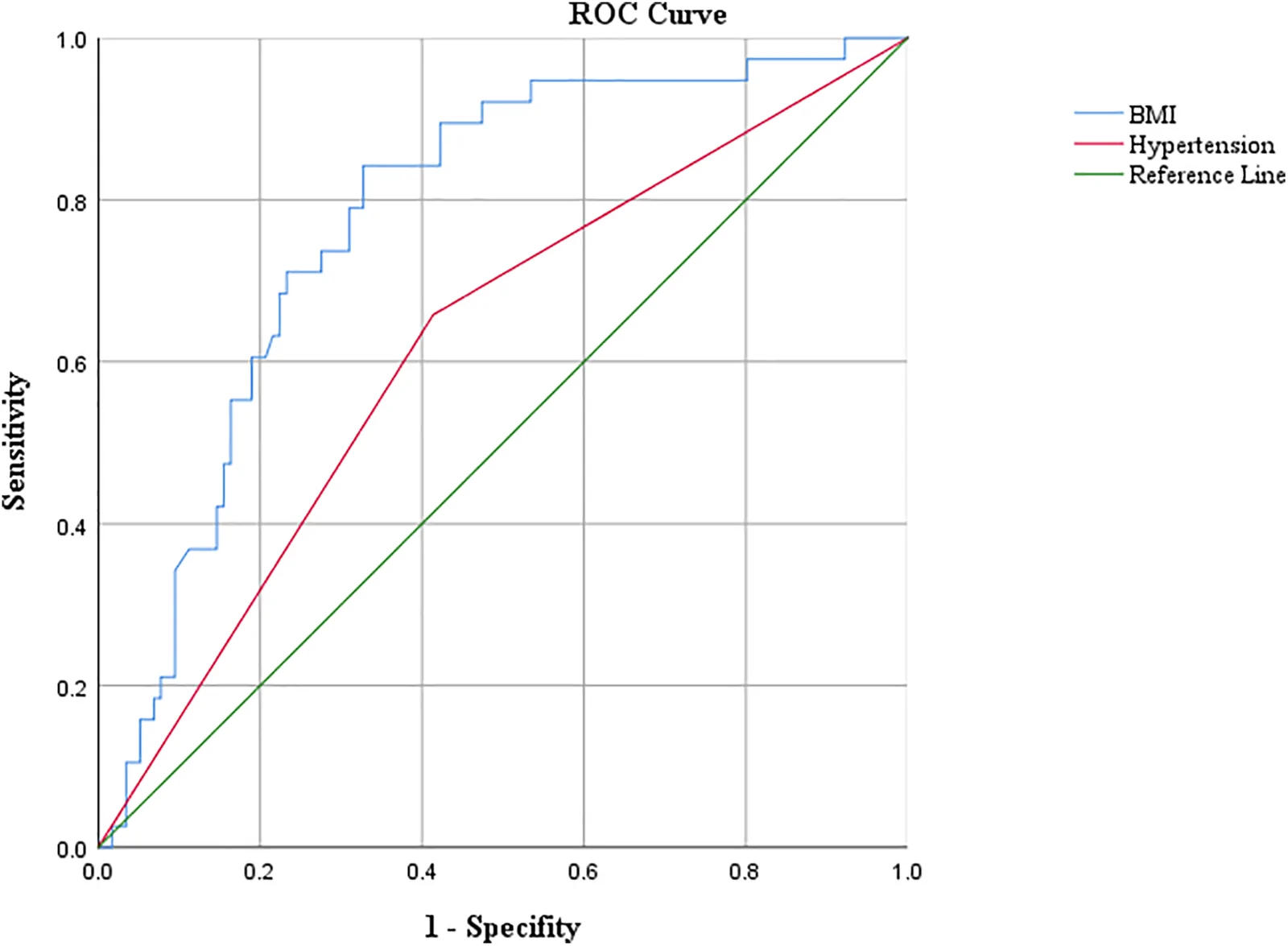
ROC curve for hypertension and BMI in high HBL group after TKA. Patients with BMI > 26.34 kg/m² are at significantly increased risk of high HBL after TKA.

ROC curve analysis was conducted to evaluate the discriminative performance of BMI and hypertension in identifying patients with high postoperative HBL. BMI demonstrated moderate discriminative ability, with an area under the curve (AUC) of 0.778 (95% CI: 0.697–0.858, P < 0.001). The optimal cutoff value determined using Youden’s index was 26.34 kg/m², yielding a sensitivity of 84.2% and a specificity of 66.4%. Internal validation using stratified 10-fold cross-validation demonstrated consistent discrimination, with a mean AUC of 0.785 ± 0.134. The cross-validated optimal BMI cutoff remained stable at 26.49 ± 0.36 kg/m², with a mean sensitivity of 78.3% and specificity of 69.0%. At this BMI threshold, the prevalence of high HBL in the cohort was 24.7%, with a positive predictive value (PPV) of 45.1% and a negative predictive value (NPV) of 92.8%. In contrast, hypertension, although significantly associated with high HBL, showed limited discriminative ability as a standalone marker, with an AUC of 0.622 (95% CI: 0.520–0.724, P = 0.024). Collectively, these findings suggest that BMI provides greater discriminatory capability than hypertension for identifying patients at increased risk of high HBL and may serve as a potential marker for perioperative risk stratification and individualized management in patients undergoing TKA. Detailed results are presented in Table 4 and illustrated in Fig 1.

**Table 4:** Predictive Performance of Independent Risk Factors for HBL.

	AUC^1^	95%CI^2^	P^*^	cut-off value	Sensitivity	Specificity
BMI(kg/m²)	0.778 (0.785 ± 0.134)^3^	(0.697,0.858)	0.000	26.34 (26.49 ± 0.36)^3^	84.2% (78.3%)^3^	66.4% (69.0%)^3^
Hypertension (Yes/No)	0.622	(0.520,0.724)	0.024	–	–	–

^1^,AUC: Area under the ROC, BMI demonstrates strong discriminative power (AUC = 0.778), substantially outperforming hypertension (AUC = 0.622), and can guide preoperative planning. ^2^,95% CI: 95% Confidence Interval;^3^,Values were obtained using 10-fold internal validation in R for BMI, including AUC, cut-off, sensitivity, and specificity, indicating model robustness;^*^,P < 0.05 was considered statistically significant.

## Discussion

In recent years, HBL has received increasing clinical attention. Multiple studies have confirmed that HBL constitutes a substantial proportion of total perioperative blood loss in orthopedic procedures [26–28]. The magnitude of HBL is influenced by a variety of factors, including patient-specific characteristics, the severity of KOA, surgical technique, and postoperative management. This study specifically focused on evaluating patient and surgical variables, and our findings identified BMI as an independent risk factor and hypertension as a related risk factor for high HBL following TKA. These results underscored the importance of comprehensive preoperative assessment and targeted perioperative management strategies to mitigate excessive blood loss and optimize surgical outcomes.

Importantly, HBL is not confined to knee arthroplasty but represents a broader concern across joint replacement procedures. Evidence from total hip arthroplasty (THA) has demonstrated similar HBL patterns with significant clinical implications. For example, Micicoi et al. have reported that HBL accounts for a major component of overall blood loss in THA, highlighting the necessity of systematic perioperative blood management strategies in both hip and knee arthroplasty [29]. This cross-procedural perspective contextualizes our findings within the broader field of arthroplasty research and emphasizes the clinical relevance of addressing HBL in TKA.

In the present study, patients in the high-HBL group exhibited a significantly higher mean BMI compared with those in the low-HBL group. This observation aligned with prior THA studies by Cai et al. and Sun et al., which have identified BMI as an independent predictor of perioperative HBL [30,31]. However, large-scale studies in TKA have reported contrasting findings: Deng et al. found no significant association between BMI and HBL [20], while Cao et al. have reported that obesity does not increase perioperative blood loss in multicenter TKA cohorts [32]. These discrepancies suggest that the impact of BMI on HBL may vary according to arthroplasty type, surgical technique, and patient population. In our cohort, the optimal BMI threshold for predicting high HBL was 26.34 kg/m², slightly above the World Health Organization’s definition of overweight (≥25 kg/m²) and just below the Asian obesity threshold (≥27.5 kg/m²). This observation enhanced the external validity of our findings by indicating that even patients classified as overweight, not yet obese by regional standards, are already at elevated risk for high HBL. Several mechanisms may underlie this association. Elevated intra-abdominal and ventilatory pressures in overweight patients can increase venous pressure, predisposing to greater bleeding [33]. Additionally, a thicker adipose layer complicates joint exposure and increases soft tissue trauma, while a larger blood volume and expanded extravascular spaces may further contribute to greater perioperative HBL [34]. At the identified BMI threshold of 26.34 kg/m², BMI demonstrated a sensitivity of 84.2% and a specificity of 66.4% for identifying patients with high HBL. The relatively high sensitivity suggests that this cutoff may help identify most patients at risk of excessive HBL, whereas the lower specificity indicates that BMI alone may not be sufficient as a definitive predictor. Notably, the negative predictive value reached 92.8%, suggesting that patients with BMI below this threshold were unlikely to develop high HBL. Conversely, the positive predictive value was 45.1%, indicating that additional clinical factors should be considered when assessing patients with elevated BMI. Therefore, BMI may be more appropriately interpreted as a practical risk stratification marker rather than an independent predictive tool. Recommended measures include: (1) Preoperative optimization: Implement structured weight reduction programs, individualized nutritional counseling, and targeted prehabilitation to enhance cardiopulmonary tolerance, optimize metabolic status, and promote wound healing; (2) Intraoperative management: Conduct meticulous hemostasis assessments before wound closure, minimize unnecessary soft tissue dissection, and reinforce electrocautery along adipose–muscle interfaces to prevent diffuse oozing and reduce intraoperative hidden bleeding; (3) Postoperative care adjustments: Extend compression dressing application, maintain cryotherapy for prolonged periods, and employ staged, progressive rehabilitation protocols to minimize early joint swelling, interstitial exudation, and secondary postoperative bleeding. Collectively, these strategies can reduce HBL, shorten recovery time, and improve overall surgical outcomes in overweight and obese patients undergoing TKA.

In our cohort, hypertension was identified as a significant independent risk factor for high HBL (OR = 2.986, P = 0.011). Mechanistically, this association is likely driven by chronic vascular remodeling, including arteriolosclerosis and endothelial dysfunction. These pathological changes diminish microvascular contractile capacity and increase capillary fragility, promoting seepage into soft tissues after tourniquet release [20,35]. However, despite this strong association, the discriminative ability of hypertension in ROC analysis was modest (AUC = 0.622). This discrepancy likely stems from two factors. First, standardized perioperative management mitigates hemodynamic fluctuations. While vascular pathology predisposes patients to bleeding, rigorous blood pressure control limits the clinical manifestation of this risk [20,31]. Second, our binary classification (presence vs. absence) did not account for disease heterogeneity. Patients with well-controlled hypertension may not exhibit the same bleeding risk as those with severe or uncontrolled disease, which dilutes predictive specificity. Clinically, these findings suggest a distinction in risk assessment. Unlike BMI, which demonstrated a relatively higher discriminative ability and an identifiable threshold, hypertension may primarily reflect underlying vascular vulnerability and highlights the importance of maintaining perioperative hemodynamic stability.

Previous studies focusing on HBL after TKA have suggested that longer operative time may be associated with increased risk of high HBL [20]. Prolonged operative time may reflect greater surgical complexity, more extensive soft tissue manipulation, and increased tissue trauma, which may subsequently enhance local inflammatory responses and microvascular injury, promoting blood sequestration within periarticular tissues and contributing to HBL [36]. In the present study, operative time was significantly longer in the high-HBL group in univariate analysis, suggesting a potential association between prolonged surgery and high HBL. However, operative time was not independently associated with high HBL after multivariable adjustment. This finding suggests that operative time may primarily reflect surgical complexity and the extent of intraoperative tissue manipulation rather than serve as an independent determinant of HBL. The attenuation of this association after adjustment indicates that the observed relationship in univariate analysis may have been influenced by multiple patient-related and perioperative factors. Although operative time alone may have limited predictive value, maintaining efficient surgical workflows, minimizing unnecessary operative prolongation, and reducing excessive soft tissue trauma may still represent important considerations for optimizing perioperative blood management after TKA.

Previous studies have suggested that tourniquet application patterns may influence HBL after TKA [37,38]. Continuous tourniquet use may reduce intraoperative blood loss but potentially increase postoperative HBL through ischemia–reperfusion injury, reactive hyperemia, and tissue-related changes after tourniquet release [39–41]. However, in the present study, tourniquet application pattern was not significantly associated with high HBL. This discrepancy may be partly explained by the standardized perioperative management in our cohort, including a consistent TXA protocol and relatively uniform surgical procedures, which may have minimized the influence of tourniquet-related factors. Therefore, although tourniquet strategies may affect perioperative blood distribution under certain circumstances, their independent contribution to high HBL appeared limited in our cohort.

Notably, despite a mean HBL of approximately 794 mL in the high-HBL group, none of the patients required blood transfusion. This observation reflected both our restrictive transfusion protocol (Hb < 70 g/L or symptomatic anemia) and the beneficial impact of enhanced recovery pathways, including the routine administration of TXA. Accumulating evidence supports the role of TXA in substantially reducing HBL following TKA. For instance, Li et al. have demonstrated that an additional postoperative intravenous dose of TXA further decreases HBL after primary TKA, while other studies have confirmed its overall effectiveness in mitigating hidden bleeding [13,16]. These findings reinforce the external validity of our results and help explain the absence of transfusion in our cohort despite substantial HBL.

We acknowledge several limitations inherent to this study. First, the single-center, retrospective design reflects the specific surgical and anesthetic protocols of our institution. While this uniformity minimizes internal confounding variables, such as surgical technique, it inevitably restricts the extrapolation of our results to broader populations. Validation through multicenter prospective cohorts is therefore necessary to confirm the generalizability of these risk factors. Second, although the sample size was adequate for the primary multivariate analysis, the uneven distribution within subgroups, specifically the predominance of female patients and the smaller proportion of hypertensive individuals, may have limited the statistical precision of effect estimates. Future studies with larger, balanced cohorts are recommended to better quantify these associations and support more granular subgroup analyses. Finally, the stratification of key predictors remained limited. Hypertension was operationalized as a binary definition rather than by severity, and BMI was analyzed without subdivision into specific obesity classes. This precluded the assessment of potential dose-dependent relationships. In addition, although the logistic regression model identified independent factors associated with high HBL, the Hosmer–Lemeshow test suggested potential limitations in model calibration, which may require further validation in larger multicenter cohorts. Future investigations incorporating detailed data on disease severity, specific BMI subcategories, and external validation cohorts would further refine the predictive accuracy and clinical applicability of risk stratification models.

## Conclusion

HBL following TKA represented a clinically significant contributor to perioperative morbidity. This study identified BMI > 26.34 kg/m² as an independent predictor of high HBL and further demonstrated that hypertension was independently associated with an increased risk of high HBL. Based on these findings, we proposed the following practical recommendations: (1) preoperative BMI screening and counseling: incorporate BMI assessment into routine preoperative evaluation to identify high-risk patients and provide tailored counseling focused on weight optimization prior to surgery; (2) Blood-conservation strategies in overweight patients: Apply enhanced TXA protocols, ensure meticulous intraoperative hemostasis, and maintain restrictive transfusion thresholds to minimize HBL; (3) Optimization of hypertension management prior to surgery: Ensure adequate blood pressure control and review antiplatelet or anticoagulant therapy in hypertensive patients to further reduce the risk of postoperative bleeding. Implementation of these measures has the potential to decrease HBL, accelerate postoperative recovery, and improve overall surgical outcomes. Future multicenter prospective studies are warranted to refine BMI-based risk thresholds and develop standardized perioperative blood management algorithms specifically tailored for overweight and hypertensive patients undergoing TKA.

## Supporting information

S1 Data Raw data. (XLSX)
